# A novel *SF3B1* variant in a patient with MDS with ring sideroblasts

**DOI:** 10.1002/jha2.500

**Published:** 2022-06-24

**Authors:** Yahya A. Al‐Ghamdi, Michael J. Kluk

**Affiliations:** ^1^ Department of Pathology and Laboratory Medicine Weill Cornell Medicine New York New York USA

1

An 80‐year‐old male was investigated for anemia. Complete blood counts showed WBC of 4.0 × 10^9^/L, hemoglobin of 92 g/L, hematocrit of 27.2%, mean corpuscular volume of 103.7 fl, red cell distribution width of 19.7%, and platelets of 140 × 10^9^/L. Aspirate smears showed 4% blasts, decreased myeloid to erythroid ratio (0.7:1), and megaloblastoid erythroid elements (>10%; Figure 1, panel A, original magnification 1000×, Wright–Giemsa stain). The iron stain showed ∼94% ring sideroblasts (Figure 1, panel B, original magnification 1000×, iron stain). The bone marrow biopsy showed a 60% cellular marrow with occasional hypolobulated megakaryocytes (>10%; Figure 1, panel C, original magnification 200×, hematoxylin and eosin stain; Figure 1, panel D, original magnification 400×, Wright–Giemsa stain). A custom next‐generation sequencing panel (45 genes, RainDance/Illumina) showed *SF3B1* c.1862A>T, p.D621V at 39.9% variant allele frequency (VAF) and *TET2* c.1862A>T, p.S1898F at 3.6% VAF. Cytogenetics showed a normal karyotype. The diagnosis of myelodysplastic syndrome (MDS) with ring sideroblasts (RS) and multilineage dysplasia (MLD) was rendered.

**FIGURE 1:**
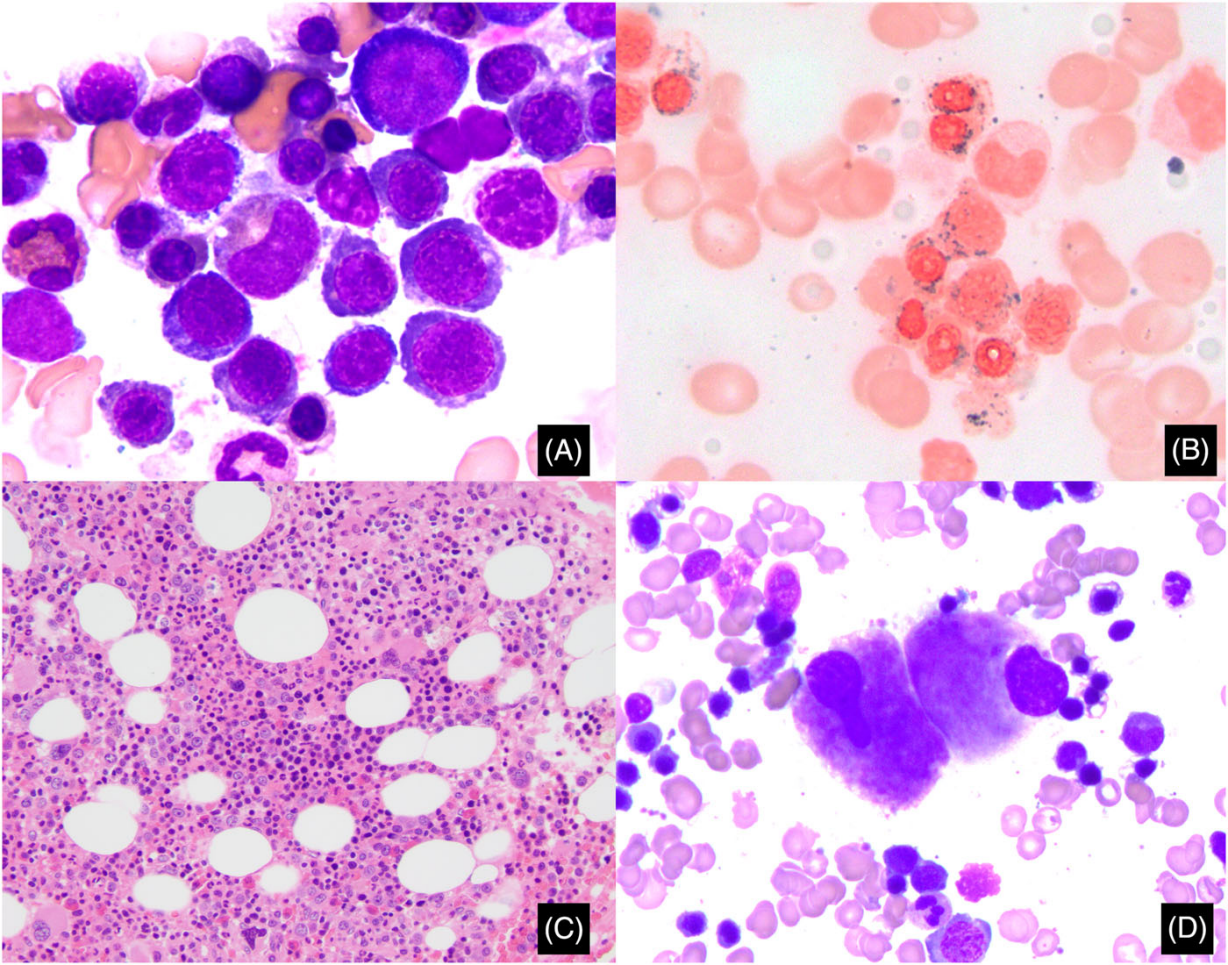
(A) Aspirate smear showing megaloblastoid erythroid precursors (original magnification 1000×, Wright–Giemsa stain). (B) Iron stain showing numerous ring sideroblasts (original magnification 1000×, iron stain). (C) Bone marrow core biopsy showing a hypercellular marrow with erythroid predominance and hypolobulated megakaryocytes (original magnification 200×, hematoxylin and eosin stain). (D) A high‐power image of hypolobulated megakaryocytes (original magnification 400×, Wright–Giemsa stain)

To our knowledge, *SF3B1* c.1862A>T, p.D621V identified in this case has never been reported to be pathogenic. This case highlights how the morphologic findings (i.e., ring sideroblasts) were very important to guide the interpretation of this novel *SF3B1* variant as pathogenic. And, in turn, the molecular evidence of an *SF3B1* mutation permitted definitive diagnosis and classification of the morphologic findings.

## CONFLICT OF INTEREST

The authors declare no conflict of interest.

## FUNDING INFORMATION

This study received no specific funding.

## Data Availability

The authors agree for data sharing and the journal policy.

